# Heat Stress-Induced Male Reproductive Dysfunction in Brandt’s Vole (*Lasiopodomys brandtii*): A Central Role for Endoplasmic Reticulum Stress

**DOI:** 10.3390/biology15171553

**Published:** 2026-09-05

**Authors:** Kang Lou, Jiaxue Jin, Yankai Yang, Xiaomeng Zhao, Lijuan Zhao, Zhiguang Chang, Senlin Li, Zhenlong Wang

**Affiliations:** 1 School of Life Sciences, Zhengzhou University, Zhengzhou 450001, China; 2 Medical School, Huanghe Science & Technology College, Zhengzhou 450063, China

**Keywords:** Brandt’s vole, climate warming, ER stress, testicular injury

## Abstract

Rising global temperatures pose a serious threat to wild small mammals’ population persistence. In this study, we investigated the molecular mechanisms underlying heat-induced testicular impairment in Brandt’s vole (*Lasiopodomys brandtii*), a dominant small mammal species of the Eurasian temperate steppe. Adult males were subjected to short-term heat exposure at 37 °C, 39 °C, and 41 °C. Heat stress at temperatures ≥ 39 °C significantly reduced the testicular index and caused histopathological damage. Integrated transcriptomic and data-independent acquisition proteomic analyses revealed significant enrichment of pathways related to endoplasmic reticulum protein processing and barrier function. Further molecular validation showed significant upregulation of key ER stress-related markers, including total eIF2α, ATF4, ATF6B, and XBP1, suggesting the potential involvement of the unfolded protein response. Together, these results identify endoplasmic reticulum stress as a key mediator of heat-induced testicular injury and highlight that 41 °C leads to marked activation of ER stress markers and severe testicular impairment in Brandt’s voles, even following short-term exposure.

## Simple Summary

Rising global temperatures pose a significant threat to the reproduction of wild small mammals. This study investigated how short-term heat exposure damages the reproductive health of male Brandt’s voles. We found that heat stress at or above 39 °C significantly damages testicular structure and reduces testicular weight. Using transcriptomic and proteomic analyses, we discovered that this heat-induced injury escalates at 41 °C, triggering a severe unfolded protein response in the testes and disrupting protein processing in the endoplasmic reticulum. These findings demonstrate that physiological impairment begins at 39 °C, while 41 °C is associated with a marked activation of cellular stress pathways (particularly ER stress) that further exacerbates testicular injury in Brandt’s voles. This study provides valuable insights into how climate change and extreme heatwaves could impact wild animal populations, even following short-term exposure.

## 1. Introduction

Rising global temperatures pose a significant threat to wild animal health and population persistence [1,2]. In male mammals, spermatogenesis is highly temperature-sensitive and requires that the scrotum be maintained approximately 2–4 °C below core body temperature. Scrotal temperature is easily elevated by the environment, which can disrupt spermatogenic homeostasis, resulting in impaired sperm quality and male infertility [3]. Brandt’s vole (*Lasiopodomys brandtii*) is a representative small mammal species of the Eurasian temperate steppe. It dominates the typical steppes of Inner Mongolia (China), Mongolia, and the Trans-Baikal region of Russia, where its population dynamics exert substantial influences on grassland structure and ecosystem functioning [4]. In the temperate steppe of Inner Mongolia, regional warming has accelerated to approximately 0.37 °C per decade, which is three times the global average [5,6]. Extreme temperatures reached 42.3 °C in 2021, as recorded by the Inner Mongolia Meteorological Observatory. In reality, ambient temperature is measured inside a shaded instrument shelter (Stevenson screen) at a standard height above the ground. However, the actual temperature within a few centimeters of the ground is significantly higher. It is precisely this near-surface microclimate temperature that influences Brandt’s vole. Climate change has been closely associated with changes in the population dynamics and geographical distribution of prairie-dwelling small mammals, including Brandt’s vole, with these changes being heavily driven by reproductive capacity [7,8,9]. Due to its high sensitivity to thermal stress, Brandt’s vole provides an ideal model for investigating how climate warming affects wild small mammals.

Endoplasmic reticulum (ER) stress signaling plays a critical role in spermatogenesis, sperm maturation, and fertilization [10]. Studies have reported that testicular dysfunction in both animal models and humans—triggered by various factors—is fundamentally driven by impaired ER homeostasis and severe ER stress-mediated unfolded protein response (UPR) signaling pathways. Notably, interventions that reduce this ER stress response have been shown to improve sperm production and quality [11]. As part of an ER-specific adaptation program, the UPR transitions from an adaptive pro-survival state into a destructive pro-apoptotic cascade under persistent stress. This shift drives male subfertility through distinct cell-specific pathways, accelerating the depletion of temperature-sensitive spermatogenic cells [12,13]. Furthermore, ER stress simultaneously degrades the tight junction proteins in Sertoli cells, thereby disrupting the integrity of the blood–testis barrier (BTB) [14].

Although heat stress is widely recognized to impair male reproductive function, the molecular mechanisms linking heat stress, ER stress, and testicular injury in wild small mammals remain poorly understood. Here, we hypothesize that climate warming impairs male reproductive function in Brandt’s voles via ER stress. Using integrated transcriptomic and proteomic analyses, we aim to elucidate the molecular basis of heat-induced testicular injury in Brandt’s voles.

## 2. Materials and Methods

### 2.1. Experimental Animals

Forty-eight adult male Brandt’s voles (45 ± 5.5 g, 9–10 weeks) were used in this study. These animals were the offspring of a wild-captured population originally sourced from the grasslands of Xilinhot, Inner Mongolia, China (43°02′–44°52′ N, 115°13′–117°06′ E). Voles were housed individually in a controlled environment maintained at a temperature of 22 ± 2 °C and relative humidity of 35–50%, under a 12:12 h light/dark cycle (lights on from 07:00 to 19:00). Animals were provided ad libitum access to standard rodent diet and water, with daily monitoring for health status and sanitary cage maintenance. All experimental procedures were approved by the Institutional Animal Care and Use Committee of Zhengzhou University (Approval No. ZZUIRB2023-008, 9 January 2023) and were conducted in strict accordance with the Guide for the Care and Use of Laboratory Animals of China.

### 2.2. Heat Stress Treatment and Sample Collection

Male Brandt’s voles were randomly assigned to four experimental groups (n = 12 per group): a control group (Ctrl) maintained at room temperature (22 ± 2 °C), and three heat stress groups subjected to temperatures of 37 °C (C37), 39 °C (C39), and 41 °C (C41), respectively. Prior to heat exposure, all animals were anesthetized via intraperitoneal injection of 5 mg/kg Xylazine (J329BA0031, Sangon Biotech, Shanghai, China). Heat stress was induced by immersing the lower abdomen and scrotum in a water bath at the corresponding temperature for 30 min as a single exposure, according to an established protocol [15]. Following treatment, animals were allowed to recover at room temperature for 24 h. The animals were subsequently euthanized, and the testes were immediately dissected and weighed. The testicular index was calculated via the following formula: Testicular Index (%) = (Testicular Weight/Body Weight) × 100. The cauda epididymis was carefully excised, rinsed in warm 0.9% NaCl solution at 37 °C, and immediately placed into 1 mL of warm saline on a 37 °C heating plate. The tissue was then gently minced with micro-scissors to allow the sperm to swim out. After a 15 min incubation to ensure proper dispersion, the fluid was thoroughly mixed, and an aliquot of the sperm suspension was collected, smeared, and stained using a commercial Diff-Quik kit (Servicebio, Wuhan, China) for comprehensive morphological analysis under a light microscope. For histological analysis, intact testes were fixed in a specialized testis fixative (Servicebio, Wuhan, China). For transmission electron microscopy analysis, testicular samples were collected immediately and fixed as described in Section 2.5. For molecular analysis, testicular tissues were snap-frozen in dry ice and stored at −80 °C until RNA and protein extraction.

### 2.3. Histological Analysis

Following fixation, testes were dehydrated and embedded in paraffin. Six micrometer-thick sections were prepared using a microtome (Thermo Fisher Scientific, San Jose, CA, USA) and subjected to standard hematoxylin and eosin (H&E) staining protocols. Slides were then scanned and documented using an Olympus VS200 SLIDEVIEW imaging system (Olympus Soft Imaging Solutions GmbH, Münster, Germany) to evaluate histological alterations.

### 2.4. Biochemical Analysis of Oxidative Stress

Testicular tissues were homogenized in an ice-water bath using 0.9% NaCl at a mass-to-volume ratio (*w*/*v*) of 1:5 to 1:10. The resulting homogenates were centrifuged at 8000× *g* for 10 min at 4 °C, and the supernatants were collected for subsequent biochemical analysis. The activities of catalase (CAT) and superoxide dismutase (SOD), as well as the concentrations of malondialdehyde (MDA) and reduced glutathione (GSH), were quantified using assay kits (Solarbio Science & Technology Co., Ltd., Beijing, China; Cat. Nos. BC4780, BC5160, BC0020, and BC1170).

### 2.5. Transmission Electron Microscopy

Testicular tissues were primarily fixed in 0.1 M cacodylate buffer (pH 7.4) containing 2.5% glutaraldehyde and 1.4% sucrose at 37 °C for 1.5 h. Samples underwent post-fixation in 1% osmium tetroxide and were subsequently dehydrated and embedded in Epon-812 resin. Ultra-thin sections (90 nm) were prepared using an ultramicrotome (Leica EM UC7, Leica Microsystems GmbH, Wetzlar, Germany) and double-stained with uranyl acetate and lead citrate. Testicular ultrastructure was visualized using a transmission electron microscope (JEM-1400, JEOL Ltd., Tokyo, Japan) operating at an acceleration voltage of 80 kV.

### 2.6. Quantitative Real-Time PCR Analysis

Total RNA was isolated from testicular tissue using the Trizol reagent (Solarbio Science & Technology Co., Ltd., China; Cat. No. P1011). RNA concentration and purity were assessed using a NanoDrop spectrophotometer (NanoDrop 2000, Thermo Fisher Scientific, Waltham, MA, USA). First-strand cDNA was synthesized using the HiScript^®^ III All-in-One RT SuperMix Perfect kit (Vazyme Biotech Co., Ltd., Nanjing, China; Cat. No. R333-01). Quantitative real-time PCR was performed using the SYBR green method on an Applied Biosystems 7500 real-time PCR system (Applied Biosystems/Thermo Fisher Scientific, Waltham, MA, USA). Species-specific primers for Brandt’s voles were synthesized by Sangon Biotech (Shanghai, China), and all primer sequences are provided in Supplementary Table S1.

### 2.7. Western Blot Analysis

Tissue samples were lysed in RIPA buffer. For each sample, 50 μg of protein was separated by SDS-PAGE gels, transferred to PVDF membranes (Milipore, Burlington, MA, USA), and blocked with 5% skimmed milk (1 h, 25 °C). Membranes were incubated with primary antibodies overnight at 4 °C, followed by HRP-conjugated secondary antibody (1 h, 25 °C). Signals were detected using an ECL kit (Huaxingbio, China) on an ImageQuant LAS 4000 mini system (GE Healthcare, Chicago, IL, USA) and quantified via ImageJ 1.43n. β-tubulin was used as the internal control. The antibodies used were: ATF4 (D320294, Sangon Biotech), XBP1 (D121401, Sangon Biotech), total eIF2α (D163768, Sangon Biotech), β-Tubulin (YM2235, ImmunoWay Biotech), and ATF6B (D223623, Sangon Biotech).

### 2.8. RNA Sequencing and Data Analysis

Total RNA was extracted from the testicular tissues of Brandt’s voles (n = 5 biological replicates per group) for RNA-seq analysis. Transcriptome sequencing was performed by Feisha Biotechnology (Wuhan, China). Raw sequence data were quality-filtered using fastp (v1.0.1) to remove low-quality reads and adapter sequences. High-quality clean reads were assembled de novo using Trinity (v2.5.1). Temporal or pattern-based gene expression profiles were analyzed using the Mfuzz R package (v2.60.0) with default fuzzy c-means clustering parameters. Differentially expressed genes (DEGs) were identified using a threshold of fold change ≥ 2 and False Discovery Rate (FDR)-adjusted *p*-value (P-adj) ≤ 0.01. Functional annotation of DEGs was conducted against multiple databases, including the NCBI non-redundant (NR) protein sequences, Gene Ontology (GO), and Kyoto Encyclopedia of Genes and Genomes (KEGG). Evolutionary genealogy of genes was assessed using Non-supervised Orthologous Groups (eggNOG), Swiss-Prot, and Pfam.

### 2.9. Liquid Chromatography–Tandem Mass Spectrometry and Data Analysis

Protein samples were prepared from the testicular tissues of Brandt’s voles (n = 3 biological replicates per group) for proteomic analysis. Based on transcriptomic screening, the C39 group was selected for subsequent proteomic analysis. Testicular protein expression profiles were characterized using data-independent acquisition (DIA) mass spectrometry performed with an Orbitrap Astral mass spectrometer (Thermo Fisher Scientific, Waltham, MA, USA) provided by SanshuBio Co. Ltd. (Shanghai, China). Samples were analyzed in DIA mode. Raw MS data were processed using DIA-NN (v1.9). Protein identifications were filtered at a 1% FDR at both the precursor and protein levels. Differentially expressed proteins (DEPs) were identified using a threshold of fold change ≥ 1.5 (or ≤0.67 for downregulation) and an unadjusted *p*-value < 0.05. DEPs were functionally annotated against GO and KEGG databases using eggNOG-mapper v2.

### 2.10. Statistical Analysis

Data are presented as the mean ± SEM. Statistical analyses were performed using GraphPad Prism (version 9.1.0, GraphPad Software, San Diego, CA, USA). Prior to parametric analysis, the normality of data distribution was assessed using the Shapiro–Wilk test, and the homogeneity of variance was evaluated using the Brown–Forsythe test. Differences among groups were evaluated using ordinary one-way ANOVA followed by Dunnett’s multiple comparisons test. A value of *p* < 0.05 was defined as the threshold for statistical significance.

## 3. Results

### 3.1. Heat Stress Impaired Testicular Histological Structure

**Figure 1:**
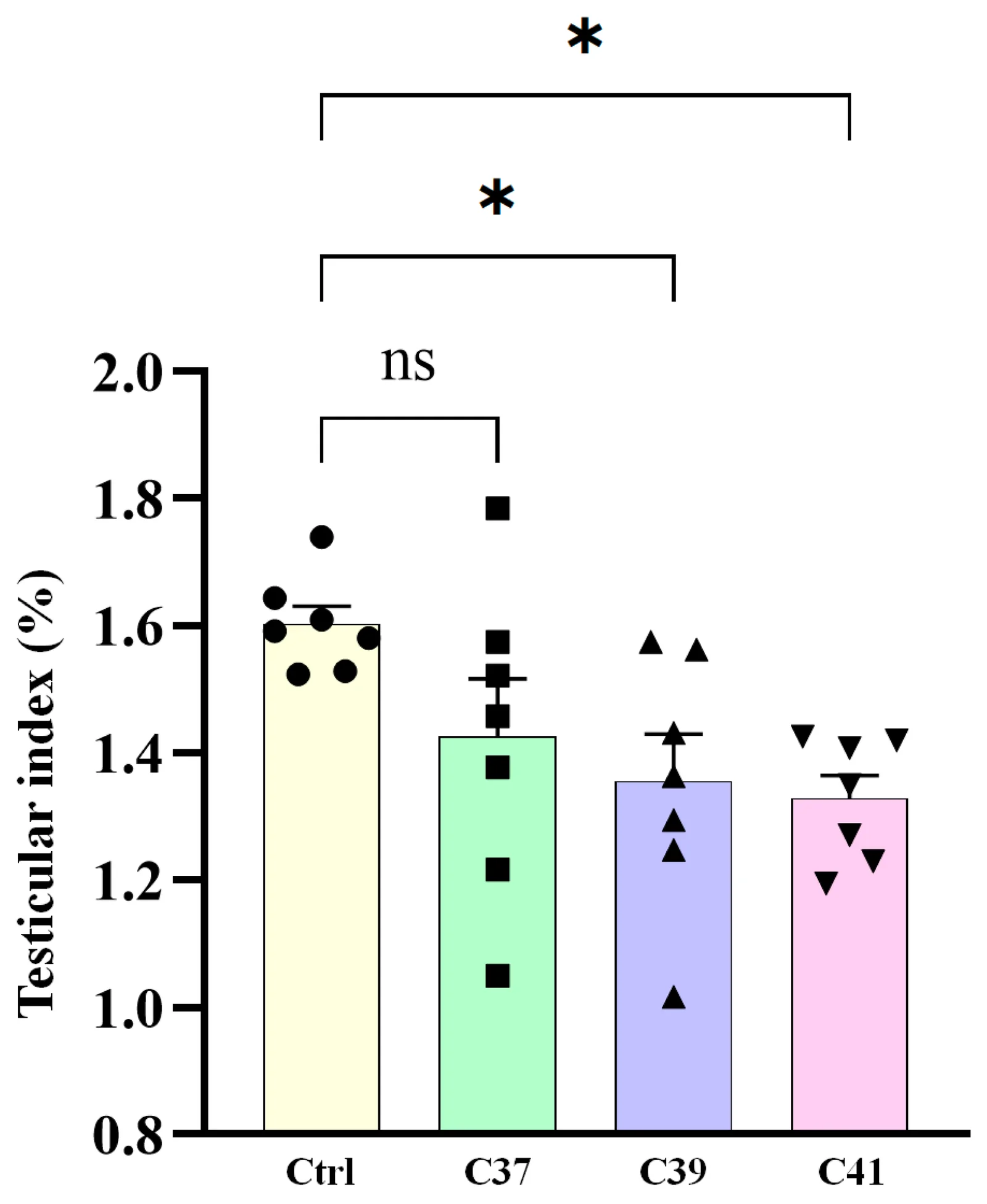
Testicular index. Data are expressed as mean ± SEM (n = 7 per group). * means *p* < 0.05. ns, no significance.

**Figure 2:**
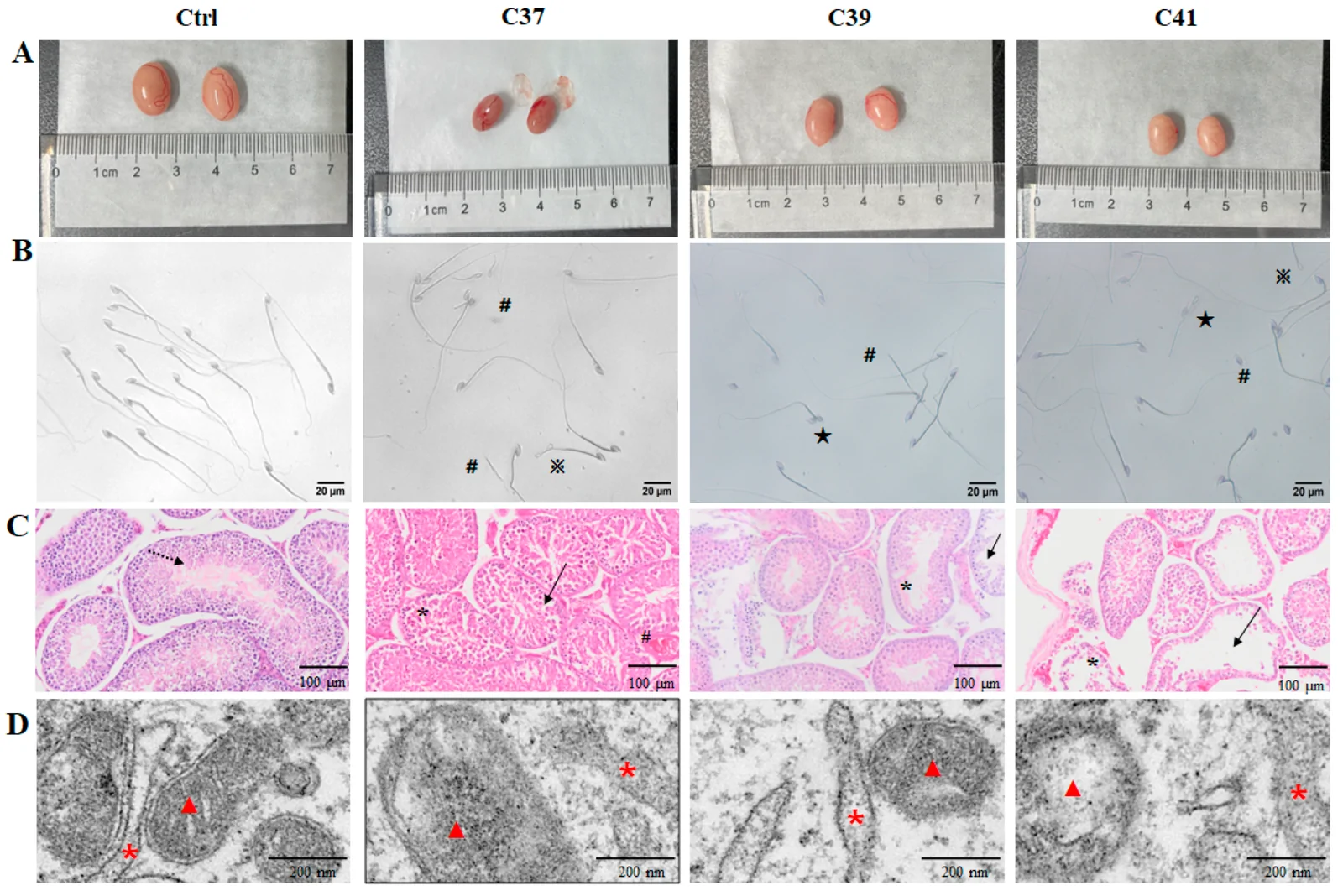
Histological evaluation of testes from heat-stressed groups. (**A**) Testicular morphology; (**B**) sperm morphology (#: decapitated spermatozoa; ※: coiled-tail; ★: multiple heads); (**C**) histological structure of testicular tissue (dotted arrow: normal seminiferous tubule with spermatozoa in lumen; #: interstitial vascular congestion; *: collapse and vacuolization of seminiferous epithelium; arrow: germ cell depletion); (**D**) ultrastructure of endoplasmic reticulum (*) and mitochondria (▲). n = 3 per group.

As illustrated in Figure 1 and Figure 2A, following heat exposure, a significant reduction in testicular index was observed at 39 °C and 41 °C (*p* < 0.05), whereas no significant change was observed in the 37 °C group. Morphological assessment of spermatozoa revealed normal structures in the Ctrl group, whereas the heat-stressed groups exhibited marked sperm abnormalities, including absent-tail, multi-headed, and coiled-tail phenotypes (Figure 2B). Histological examination of the Ctrl group revealed a healthy testicular architecture characterized by well-organized spermatogonia layers and abundant luminal spermatozoa. In contrast, the heat-stressed groups displayed progressive morphological deterioration, including vascular congestion, irregular seminiferous tubule contours, and a disrupted spermatogenic epithelium (Figure 2C). Furthermore, ultrastructural analysis via TEM demonstrated that heat stress induced severely impaired mitochondrial integrity and pronounced swelling of the ER (Figure 2D).

### 3.2. Heat Stress Induced Oxidative Stress in Testes

**Figure 3:**
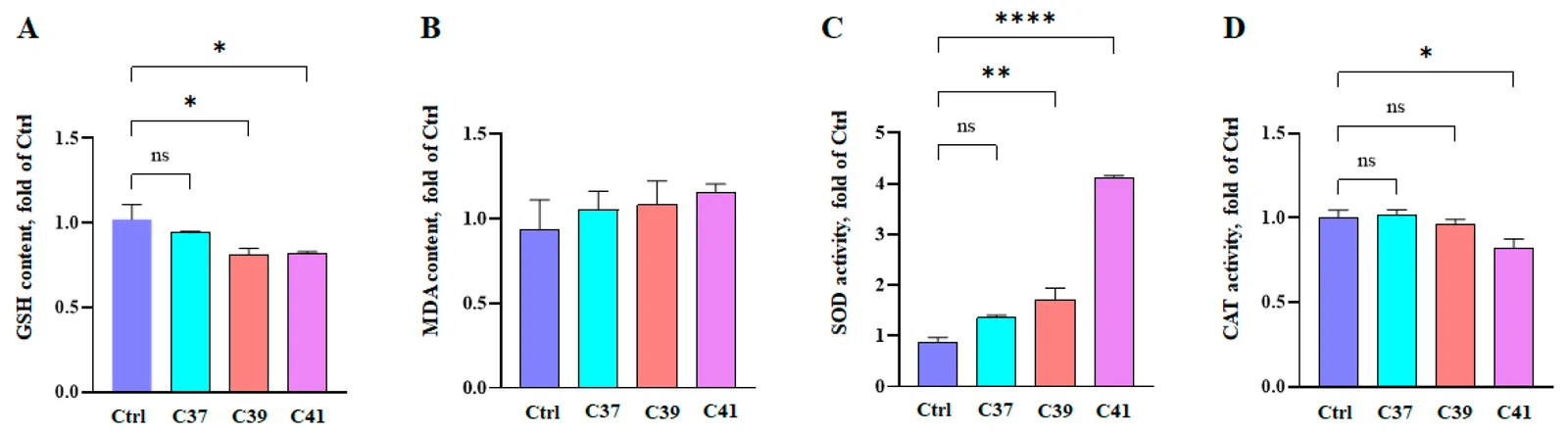
The concentrations of GSH (**A**) and MDA (**B**) and activities of SOD (**C**) and CAT (**D**) in the testicular tissue of Brandt’s voles. Data are expressed as mean ± SEM (n = 9 per group). Absence of marks or ns means no significance, * means *p* < 0.05, ** means *p* < 0.01, **** means *p* < 0.0001.

The effects of heat stress on the antioxidant capacity and lipid peroxidation of testes are summarized in Figure 3. Compared to the Ctrl group, the C39 and C41 groups showed significantly reduced GSH contents (*p* < 0.05), whereas no significant alterations were observed in the C37 group (Figure 3A). Conversely, SOD activity was upregulated in the C39 and C41 groups (*p* < 0.05) but remained unchanged under the 37 °C treatment (Figure 3C). Interestingly, MDA levels showed no fluctuation across all heat-stressed groups (*p* > 0.05; Figure 3B). Regarding CAT activity, only the C41 group showed a reduction (*p* < 0.05) (Figure 3D).

### 3.3. Heat Stress Disturbed the Barrier Function in Testes

**Figure 4:**
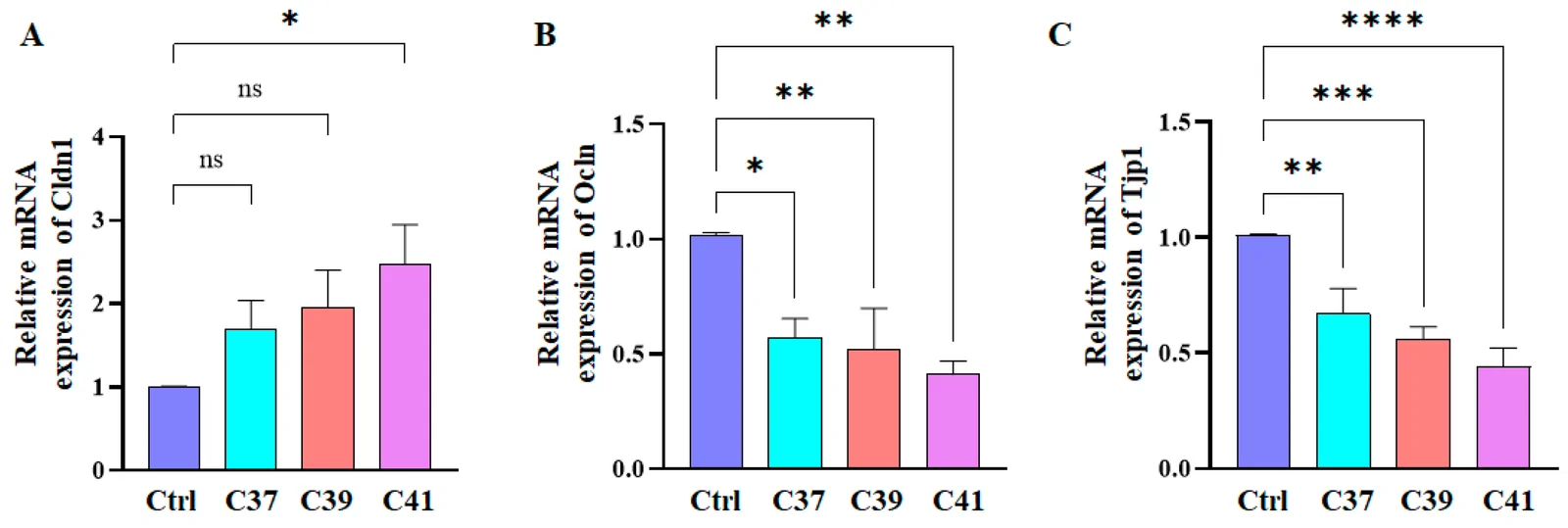
mRNA levels of *Cldn1* (**A**), *Ocln* (**B**), and *Tjp1* (**C**) in the testicular tissue of Brandt’s voles. Data are expressed as mean ± SEM (n = 8 per group). ns means no significance, * means *p* < 0.05, ** means *p* < 0.01, *** means *p* < 0.001, **** means *p* < 0.0001.

Compared to the Ctrl group, claudin-1 (*Cldn1*) mRNA abundance remained unchanged in the C37 and C39 groups, but was significantly upregulated in the C41 group (*p* < 0.05; Figure 4A). In contrast, heat stress induced a robust inhibitory effect on the expression of occludin (*Ocln*) and tight junction protein 1 (*Tjp1*) (*p* < 0.05; Figure 4B,C).

### 3.4. Heat Stress Induced ER Stress in Testes

**Figure 5:**
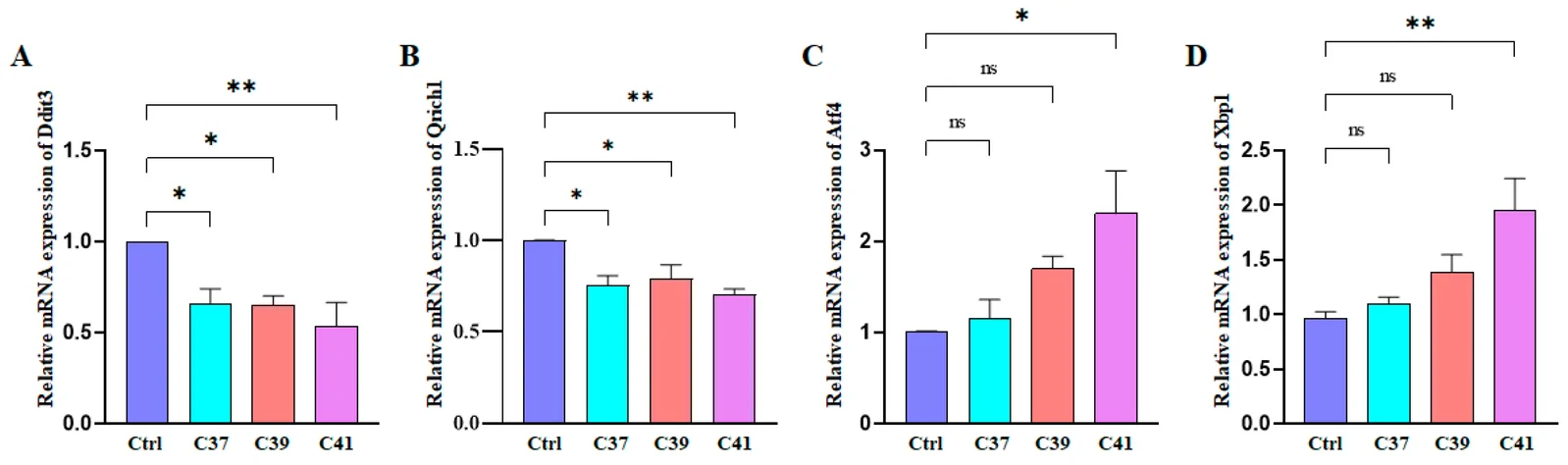
mRNA levels of *Ddit3* (**A**), *Qrich1* (**B**), *Atf4* (**C**), and *Xbp1* (**D**) in the testicular tissue of Brandt’s voles. Data are expressed as mean ± SEM (n = 8 per group). Absence of marks or ns means no significance, * means *p* < 0.05, ** means *p* < 0.01.

**Figure 6:**
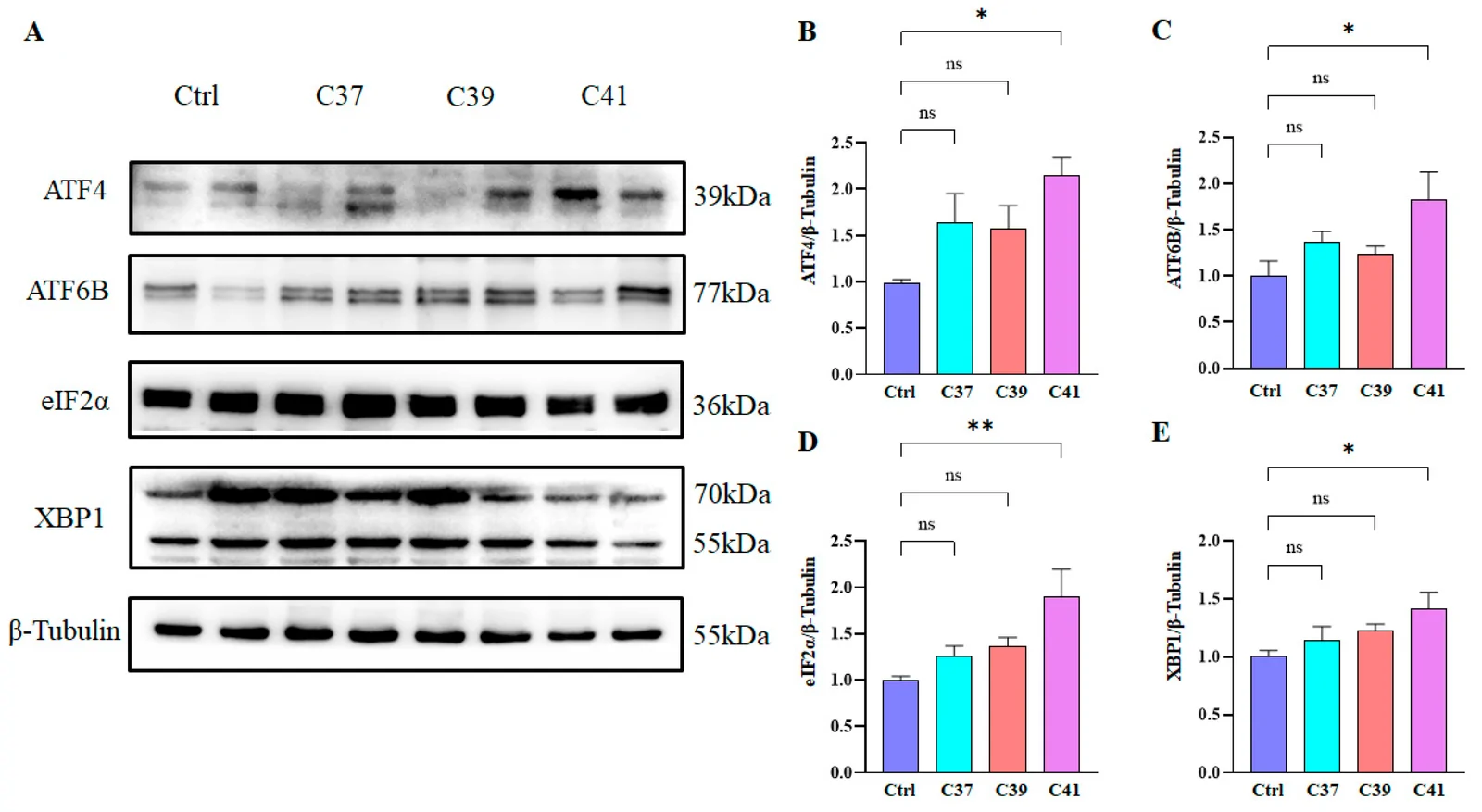
Analysis of ER stress-related protein expression in testicular tissue of Brandt’s voles. Representative Western blot images of ATF4, ATF6B, total eIF2α and XBP1 (**A**). Quantitative analysis of relative protein levels for ATF4 (**B**), ATF6B (**C**), total eIF2α (**D**) and XBP1 (**E**) in the testicular tissue of Brandt’s voles. Data are expressed as mean ± SEM (n = 6 per group). ns means no significance, * means *p* < 0.05, ** means *p* < 0.01. Please refer to Figure S10 for the original Western blot images.

The expression profiles of key ER stress markers were evaluated at both the transcriptional (Figure 5) and translational (Figure 6) levels. Unexpectedly, heat stress resulted in a downregulation of DNA damage-inducible transcript 3 (*Ddit3*) and glutamine-rich 1 (*Qrich1*) mRNA abundance (*p* < 0.05; Figure 5A,B). In contrast, the mRNA levels of activating transcription factor 4 (*Atf4*) and X-box binding protein 1 (*Xbp1*) were significantly upregulated exclusively in the C41 group, whereas no significant changes were observed in the C37 and C39 groups (Figure 5C,D).

To validate these findings, the protein abundance was analyzed via Western blotting (Figure 6A). Consistent with the transcriptional data, the protein expressions of ATF4, ATF6B, total α-subunit of eukaryotic initiation factor 2 (eIF2α), and XBP1 were exclusively elevated in the C41 group (*p* < 0.05; Figure 6B–E). Similar to the mRNA patterns, heat stress at 37 °C and 39 °C did not induce a significant increase in this protein abundance. This consistent, temperature-selective protein activation further confirms that 41 °C represents a critical molecular threshold for testicular ER stress.

### 3.5. Results of RNA-Sequencing Data Filtering

Following stringent quality control, the proportion of high-quality filtered reads ranged from 99.92% to 99.93% (Supplementary Table S2). The sequencing depth yielded between 57.02 M and 91.85 M clean reads per sample, with a minimum Q30 score of 92.97%. A total of 673,474 transcripts were identified, and the average mapping rate of reads to the assembled reference transcriptome was 86.66% (Supplementary Table S3 and Table S4).

### 3.6. Functional Annotation

A total of 350367 unigenes were successfully annotated in at least one database (Supplementary Table S5). Specifically, 63,463 unigenes were identified in the NCBI NR database, followed by 60,107 in Swiss-Prot, and 59,484 in Pfam. Furthermore, functional classification and pathway analysis assigned 57,631 unigenes to GO terms, 43,964 to eggNOG clusters, and 40,680 to KEGG pathways. Specialized annotations included 12,637 unigenes in TMHMM, 9130 in SignalP, and 3271 in the CAZy database.

### 3.7. Identification of Differentially Expressed Genes

In the C37 group, 1344 DEGs were identified, comprising 645 upregulated and 699 downregulated genes. The C39 group exhibited the highest transcriptional response with 3248 DEGs (1876 upregulated; 1372 downregulated), while the C41 group contained 1580 DEGs (763 upregulated; 817 downregulated) (Supplementary Figure S1A). Overlap analysis via a Venn diagram revealed a core set of 340 common DEGs consistently altered across all three heat treatments (Supplementary Figure S1B). The distribution and significance of these expression profiles were further visualized through volcano plots for each pairwise comparison (Supplementary Figure S1C–E).

### 3.8. DEG Cluster Analysis Based on Temperature Course

**Figure 7:**
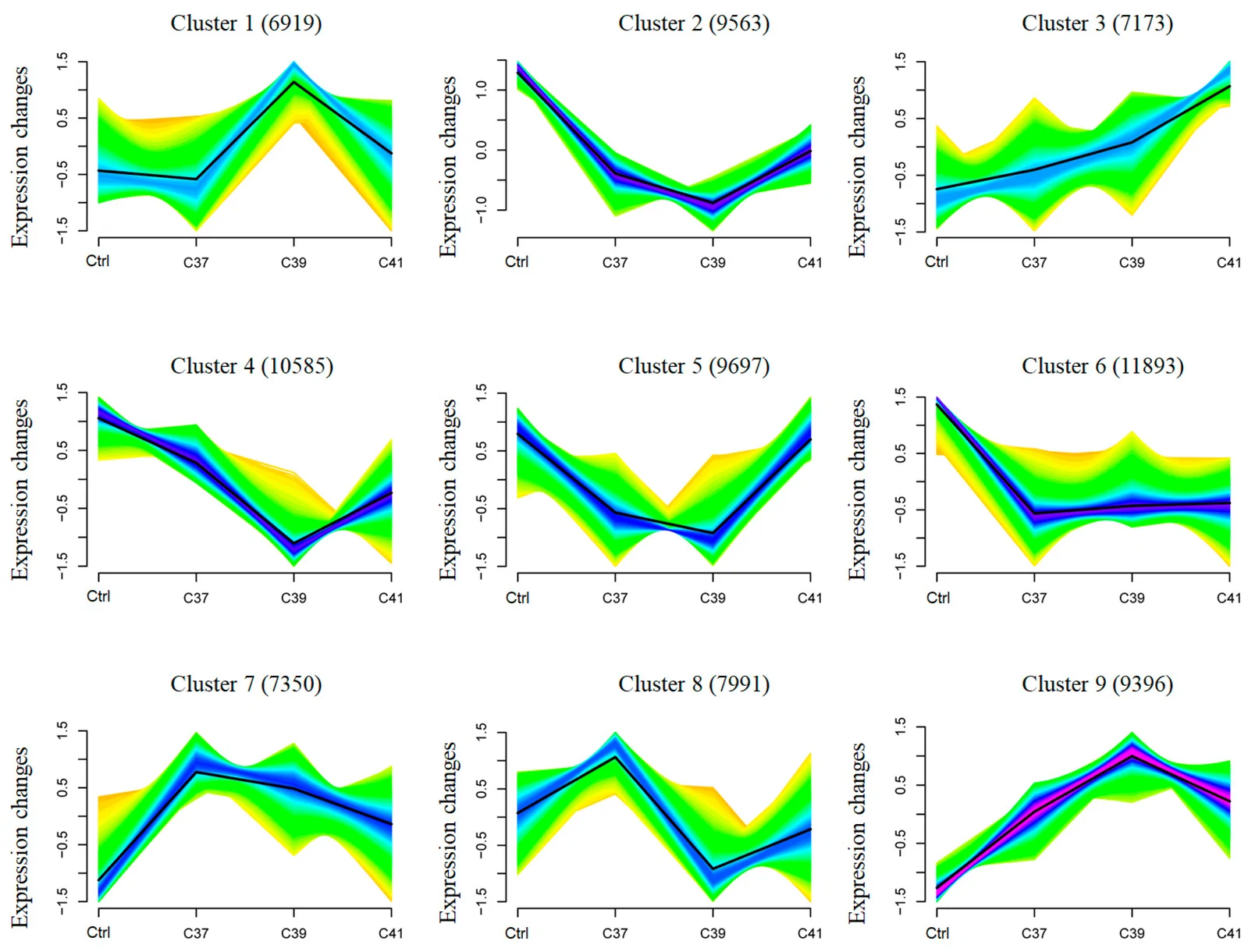
Clusters of DEGs in Ctrl and different exposed-temperature groups.

As shown in Figure 7, these clusters ranged in size from 6919 to 11,893 genes. Cluster 1 genes (6919 genes) remained at basal levels at 37 °C but showed a transient peak at 39 °C before declining at 41 °C. In contrast, genes in clusters 2, 4, and 5 exhibited V-shaped trajectories, characterized by a sharp downregulation at 37 °C or 39 °C followed by a robust induction at 41 °C. Cluster 3 genes demonstrated a progressive, temperature-dependent upregulation throughout the 37–41 °C course. Conversely, cluster 6 genes were consistently repressed across all thermal treatments. Clusters 7 and 9 displayed inverted V-shaped profiles, with marked transcriptional enhancement at 37 °C or 39 °C followed by a subsequent reduction. Finally, cluster 8 was characterized by an initial upregulation at 37 °C followed by a decrease at 39 °C.

### 3.9. GO and KEGG Enrichment Analysis of DEGs

GO analysis revealed that 833, 1734, and 995 DEGs were categorized into 54, 53, and 52 functional subcategories in the C37 (Supplementary Figure S2A), C39 (Supplementary Figure S2B), and C41 (Supplementary Figure S2C) groups, respectively. Predominantly enriched terms included “response to stimulus”, “membrane part”, and “antioxidant activity”. KEGG pathway mapping further identified 1311, 2412, and 1536 DEGs associated with diverse biological functions. These were classified into six primary KEGG categories, with the highest DEG distribution observed in environmental information processing, organismal systems, and human diseases (Supplementary Figure S3).

### 3.10. Validation of DEGs with qRT-PCR

To verify the accuracy of the RNA-sequencing findings, 15 representative DEGs involved in ER stress, inflammation, protein quality control, and metabolic regulation were selected for qRT-PCR validation (Supplementary Figure S4). Consistent with the transcriptomic profiles, the mRNA expression levels of CCAAT/enhancer binding protein delta (*Cebpd*), suppressor of cytokine signaling 3 (*Socs3*), heat shock protein beta-1 (*Hspb1*), activating transcription factor 3 (*Atf3*), membrane-associated ring-CH-type finger 10 (*Marchf10*), spermatogenesis-associated 21 (*Spata21*), meiosis-specific nuclear structural 1 (*Mns1*), SEC11 homolog A (*Sec11a*), coiled-coil domain containing 33 (*Ccdc33*), prostaglandin-endoperoxide synthase 2 (*Ptgs2*), IQ motif and ubiquitin domain-containing gene (*Iqub*), and uromodulin (*Umod*) were significantly upregulated across the heat stress cohorts. Conversely, patatin-like phospholipase domain-containing 3 (*Pnpla3*), GLI Family Zinc Finger 1 (*Gli1*), and aquaporin 2 (*Aqp2*) exhibited marked downregulation following thermal exposure. Although the magnitude of fold changes varied slightly between the two platforms, the directional trends of gene expression were highly concordant, exhibiting a strong Pearson correlation (r = 0.83, *p* < 0.001) across the 15 genes. These results provide robust validation of the transcriptomic data and confirm the reliability of the sequencing-based expression profiles.

### 3.11. Protein Identification and Quantification

A total of 5808 proteins were identified with high confidence across three biological replicates (Supplementary Table S6).

Principal component analysis (PCA) revealed that the first two components accounted for 56.3% of the total variance, with PC1 alone explaining 36.0% of the variance and clearly separating the Ctrl and C39 groups (supplementary Figure S5).

**Figure 8:**
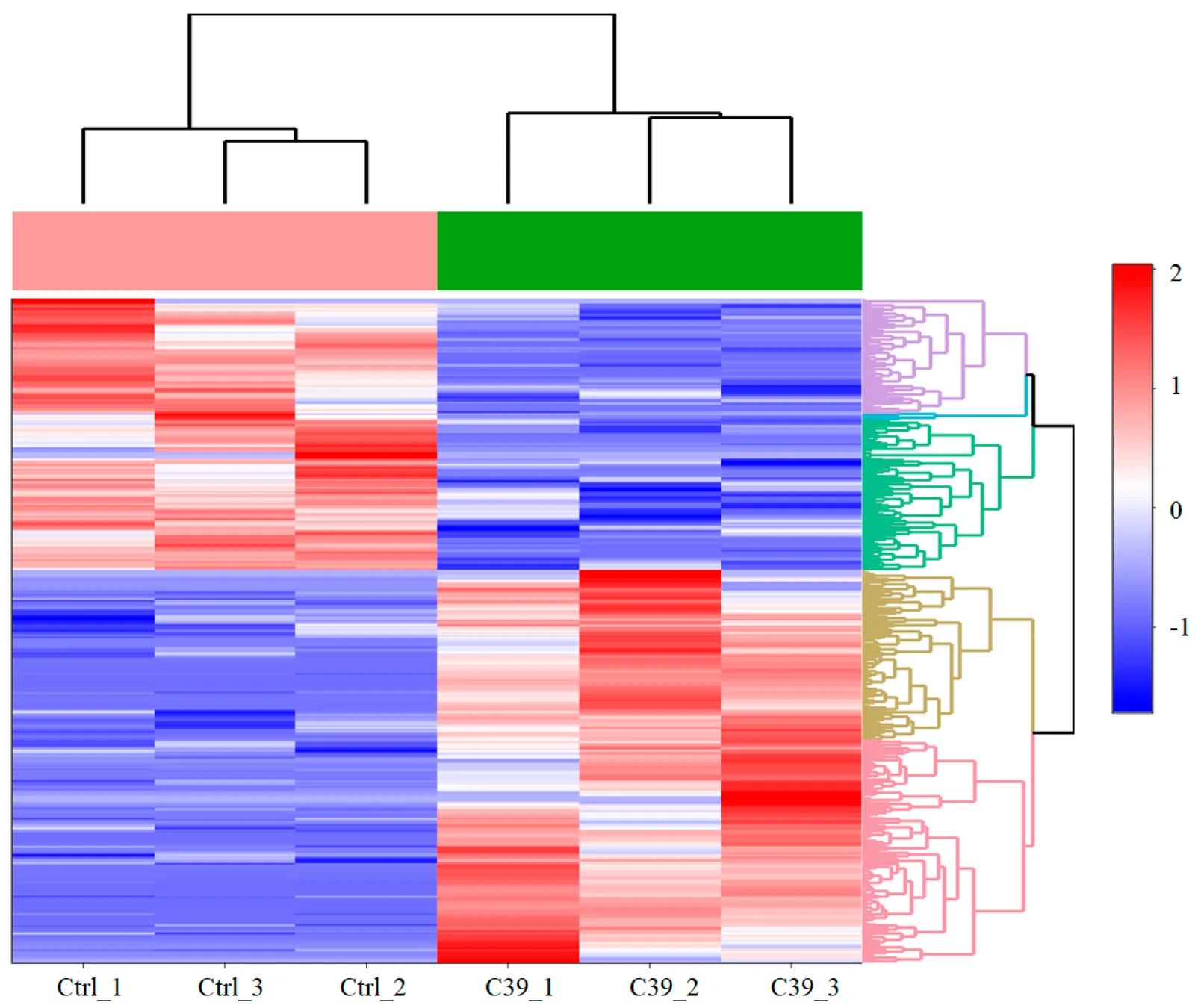
Heatmap illustrating the differentially expressed proteins (DEPs) between the Ctrl and C39 groups.

Hierarchical clustering analysis further confirmed these results (Figure 8).

A total of 394 DEPs were identified between the two groups (Supplementary Figure S6). Among these, 233 proteins were upregulated and 161 proteins were downregulated.

### 3.12. Functional Categorization Analysis

The DEPs were predominantly localized to the protoplasm, cytoplasm, cytoplasmic granule lumen, cytoplasmic granule membrane, and cytoplasmic granule (Supplementary Figure S7).

Within the biological process category, reproduction (GO:0000003) was the most highly represented term, containing 39 DEPs (with a fold enrichment of 0.18). Cellular component analysis revealed high enrichment in the nuclear chromosome, Golgi membrane, and ubiquitin ligase complexes. In the molecular function category, proteins associated with nucleotide binding and transcription cis-regulatory region binding predominated (Supplementary Figure S8).

KEGG pathway mapping further contextualized the proteomic response to heat stress (Supplementary Figure S9A,B). Significant enrichment was observed in the MAPK signaling pathway and tight junctions. Notably, stratified analysis of directional expression revealed that Heat Stress-Up DEPs were primarily associated with protein processing in the ER, neurotrophin signaling pathway, and Salmonella infection pathways. Conversely, Heat Stress-Down DEPs were predominantly enriched in pathways related to aldosterone-regulated sodium reabsorption and various cancer-related signaling modules (Supplementary Figure S9C).

## 4. Discussion

As a representative steppe rodent, Brandt’s vole provides an ideal model for examining how climate warming compromises the fertility of wild small mammals [16]. Our findings highlight ER stress as a potential key mediator of heat-induced testicular injury in Brandt’s voles. These findings establish a plausible mechanistic framework linking climate warming to male reproductive vulnerability in wild small mammals. Spermatogenesis is strictly temperature-dependent, and germ cells with high mitotic activity are particularly vulnerable to heat stress [17]. Consistent with previous studies [18,19,20], we observed that in Brandt’s voles, a single 30 min heat exposure at 39 °C or 41 °C was sufficient to markedly reduce the testicular index, induce structural sperm abnormalities, and cause widespread degeneration of the seminiferous epithelium (Figure 1 and Figure 2A–C). Heat stress is known to exacerbate ROS production, leading to oxidative stress when antioxidant defenses are overwhelmed. Although a slight upward trend was observed in MDA levels across the heat-stressed groups, it was not statistically significant (Figure 3B). Heat exposure caused a marked depletion of GSH (Figure 3A), reflecting its rapid consumption in ROS scavenging and impaired redox homeostasis [21]. In parallel, SOD activity increased, whereas CAT activity declined, indicating a potential disruption of the antioxidant cascade (Figure 3C,D). As SOD converts superoxide radicals into H_2_O_2_, reduced CAT activity would limit H_2_O_2_ detoxification, potentially favoring its accumulation. Given that H_2_O_2_ is a potent inducer of testicular cell apoptosis under heat stress, this imbalance likely underlies the reduced testicular index and histopathological damage observed at 39 °C and 41 °C [22]. Although direct measurements of H_2_O_2_ levels and specific apoptotic markers were not performed in this study, our proposed pathway provides a plausible hypothesis that warrants direct validation in future investigations. The BTB, formed by specialized junctions between Sertoli cells, is essential for maintaining the microenvironment required for spermatogenesis. In Brandt’s voles, heat stress markedly suppressed the expression of *Ocln* and *Tjp1*, consistent with Sertoli cell dysfunction and BTB disruption reported in other rodent models (Figure 4B,C) [23]. Notably, *Cldn1* expression was significantly upregulated (Figure 4A). We hypothesize that *Cldn1* upregulation in Brandt’s voles might reflect a potential cytoprotective but ultimately insufficient compensatory response to counteract BTB breakdown under heat stress. However, direct functional evidence for this role is currently lacking, and this proposed hypothesis warrants further experimental validation.

To comprehensively characterize the testicular response to heat stress, we integrated transcriptomic and proteomic analyses. Transcriptome profiling revealed extensive heat-induced gene reprogramming, with functional enrichment predominantly associated with the ER and protein proteostasis pathways, including folding, sorting, and degradation (Supplementary Figure S2). These transcriptional signatures were supported by ultrastructural evidence of ER swelling, indicating pronounced ER perturbation following heat exposure. Given the cellular heterogeneity of the testis, DIA-based quantitative proteomics was employed to resolve the executive molecular responses, focusing on the C39 group as it exhibited the most pronounced transcriptional shift compared to the C37 and C41 groups (Supplementary Figure S1A). Proteomic analysis identified differentially expressed proteins enriched within the endomembrane system and inner cell membranes, with functional annotations highlighting roles in reproduction and stress adaptation (Supplementary Figures S8 and S9). The strong consistency among transcriptomic, proteomic, and ultrastructural evidence highlights ER stress as a potential key mediator of heat-induced testicular injury in Brandt’s voles, providing a plausible mechanistic framework for the reproductive impairment observed under heat stress.

The ER is central to cellular proteostasis, maintaining an oxidative environment essential for protein folding and post-translational modification. Heat-induced ROS impair ER folding capacity, while the accumulation of misfolded proteins further amplifies ROS generation, establishing a self-reinforcing cycle of cellular stress [24,25]. To counteract this stress, cells activate the UPR, a conserved signaling network that initially limits protein synthesis and enhances chaperone expression (Figure 6) [26]. The downregulation of *Qrich1* and the pro-apoptotic factor *Ddit3* may suggest the activation of early protective mechanisms of the UPR (Figure 5A,B) [27,28]. Alternatively, this downregulation could also reflect a blunted or incomplete cellular stress response in the testicular tissue, an alternative interpretation that cannot be entirely ruled out without direct measurements of apoptotic markers. Regardless of the underlying mechanism, the downregulation of Ddit3 suggests that germ cell loss in heat-stressed Brandt’s voles may proceed via alternative pathways, such as IRE1-dependent RNA decay or mitochondria-mediated apoptosis [29,30]. Because germ cells constitute the vast majority of testicular volume, their rapid and massive apoptosis within hours of heat exposure directly accounts for the significant and rapid reduction in the testicular index observed within 24 h post-treatment.

This study demonstrates that heat exposure severely disrupts testicular function in Brandt’s voles, leading to seminiferous degeneration and impaired spermatogenesis, and increased sperm morphological abnormalities. Our integrated transcriptomic, proteomic, and ultrastructural findings consistently suggest that ER stress is a potential key mechanism driving heat-induced testicular injury. Although proteomic profiling was performed at 39 °C to capture the peak transcriptional response, the observed ER morphological swelling alongside the subsequent upregulation of related markers (total eIF2α, ATF4, ATF6B, and XBP1) suggests that ER stress responses are further heightened under severe heat stress at 41 °C. These findings highlight that while cellular defenses are mobilized early, 41 °C represents a severe stress condition that strongly activates ER stress markers and likely overwhelms protective compensation, leading to profound reproductive vulnerability in Brandt’s voles even after short-term exposure.

Several limitations in this study warrant mention. First, localized scrotal heating does not fully replicate a true, whole-body heat-wave scenario. Additionally, while the procedural intraperitoneal injection anesthesia could theoretically introduce localized tissue irritation, this factor was strictly controlled across all groups, and control animals exhibited healthy testicular profiles. Second, our study demonstrates a strong temporal association between ER stress activation and testicular injury, but lacks direct interventional evidence—such as pharmacological inhibition or induction of ER stress—to establish absolute causality. Third, we evaluated testicular parameters only at a single 24 h recovery timepoint, which limits our ability to distinguish transient physiological disruption from progressive, permanent injury. Fourth, our proteomic profiling was restricted to a single temperature group (39 °C), which may not capture the full protein expression dynamics operating at 41 °C. Lastly, while sequence homology and molecular weight specificity support our results, independent antibody validation was not performed for this non-model species. Future studies utilizing whole-body exposures, multi-timepoint recovery designs, and functional interventions will provide deeper insights into these mechanisms.

## 5. Conclusions

This study demonstrates that short-term heat exposure triggers a distinct, temperature-dependent progression of testicular impairment in male Brandt’s voles. Specifically, exposure at or above 39 °C represents the physiological threshold that severely impairs testicular structure, reduces the testicular index, and increases sperm morphological abnormalities, accompanied by germ cell degeneration and alterations in BTB-related gene expression. In contrast, 41 °C exposure was associated with marked activation of key ER stress markers (ATF4, ATF6B, total eIF2α, and XBP1), indicating severe cellular stress that likely exceeds protective compensation. Together, these findings provide an important molecular and phenotypic framework suggesting a potential link between climate warming and male reproductive vulnerability in wild small mammals. While our localized, acute (30 min) exposure paradigm under anesthesia does not fully replicate the chronic, whole-body heatwaves experienced in the wild, it represents an ecologically relevant simulation for burrow-dwelling rodents like Brandt’s voles. These voles behaviorally thermoregulate by staying in cool underground burrows and are exposed to extreme surface heat primarily during short foraging excursions. Our results demonstrate that even a single 30 min thermal exposure—mimicking a brief foraging trip—is sufficient to induce testicular injury. Thus, this study offers essential insights into how intensifying global heatwaves may threaten wild rodent populations by targeting male reproductive function.

## Data Availability

The mass spectrometry proteomics data have been deposited in the ProteomeXchange Consortium (https://proteomecentral.proteomexchange.org, accessed on 1 September 2026) via the iProX partner repository with the dataset identifier PXD073388. Reviewers can access the data using the following link: https://www.iprox.cn/page/PSV023.html;?url=17691574457356iDQ (accessed on 1 September 2026); password: 6fvL. The transcriptomic raw reads have been deposited in the NCBI Sequence Read Archive (SRA) under BioProject accession number PRJNA1399431. These data are currently private for peer review. Reviewers can access the data via the following link: https://dataview.ncbi.nlm.nih.gov/object/PRJNA1399431?reviewer=kgqqjjc63p3n0rhvnsk17h9j7c (accessed on 1 September 2026). All data will be released to the public upon the formal publication of this study.

## Supplementary Materials

The following supporting information can be downloaded at https://www.mdpi.com/article/10.3390/biology15171553/s1. Figure S1. Analysis of DEGs. Bar plot showing the number of up-regulated and down-regulated DEGs in C37, C39 and C41 groups compared to the Ctrl group (A). Venn diagram of DEGs across different comparisons (B). Volcano plot of DEGs in the Ctrl-vs-C37 (C), Ctrl-vs-C39 (D), and Ctrl-vs-C41 (E) comparison. Figure S2. Gene ontology (GO) enrichment analysis of DEGs in pairwise comparisons of Ctrl and different exposed temperature. (A) Ctrl-vs-C37, (B) Ctrl-vs-C39, (C) Ctrl-vs-C41. Figure S3. Kyoto Encyclopedia of Genes and Genomes (KEGG) analysis of DEGs in pairwise comparisons of Ctrl and different exposed temperature. (A) Ctrl-vs-C37, (B) Ctrl-vs-C39, (C) Ctrl-vs-C41. Figure S4. Comparison of gene expression data between RNA-Seq and qRT-PCR. Figure S5. Principal component analysis (PCA) of the proteomic profiles in the Ctrl and C39 groups. Figure S6. Volcano plot showing the distribution of differentially expressed proteins (DEPs) between the Ctrl and C39 groups. DEPs were identified using a threshold of fold change ≥ 1.5 (or ≤ 0.67 for downregulation) and an unadjusted *p*-value < 0.05. Figure S7. Subcellular localization of differentially expressed proteins (DEPs) in the Ctrl vs. C39 groups. Figure S8. Overview of Gene Ontology (GO) functional annotation and enrichment analysis for significant DEGs. Panels show a summary of GO terms (A) and specific enrichment results for Biological Process (BP) (B), Cellular Component (CC) (C), and Molecular Function (MF) (D). Figure S9. Bar chart illustrating the distribution of DEPs across major functional categories classified by KEGG level 2 annotations (A). Dot plot showing specific enriched pathways (level 3) identified in the comparison between the Ctrl and C39 groups (B). Butterfly plot detailing the differential enrichment of level 3 KEGG pathways for both upregulated and downregulated proteins (C). Figure S10. Original Western blot images. Table S1. Sequences of primers used in this study. Table S2. Results of RNA sequencing data filtering. Table S3. Transcript assembly length distribution. Table S4. Read alignment to a reference sequence. Table S5. Unigenes annotations. Table S6. Overview of protein and peptide identification and quantification.

## Abbreviations

*Aqp2*	aquaporin 2
*Atf3*	activating transcription factor 3
*Atf4*	activating transcription factor 4
ATF6B	activating transcription factor 6B
BTB	blood–testis barrier
CAT	catalase
*Ccdc33*	coiled-coil domain containing 33
*Cebpd*	CCAAT/enhancer binding protein delta
*Cldn1*	claudin-1
Ctrl	control
C37	37 °C
C39	39 °C
C41	41 °C
*Ddit3*	DNA damage-inducible transcript 3
DEPs	differentially expressed proteins
DIA	data-independent acquisition
eggNOG	Evolutionary Genealogy Of Genes: Non-Supervised Orthologous Groups
ER	endoplasmic reticulum
*Gli1*	Gli Family Zinc Finger 1
GO	Gene Ontology
GSH	reduced glutathione
H&E	hematoxylin and eosin
H_2_O_2_	hydrogen peroxide
*Hspb1*	heat shock protein beta-1
Iqub	iq motif and ubiquitin domain-containing gene
KEGG	Kyoto Encyclopedia of Genes and Genomes
LC-MS/MS	liquid chromatography–tandem mass spectrometry
Marchf10	membrane-associated ring-CH-type finger 10
MDA	malondialdehyde
Mns1	meiosis-specific nuclear structural 1
*Ocln*	occludin
$O_{2}^{•-}$	superoxide anion
PCA	principal component analysis
PC1	primary component
Pnpla3	patatin-like phospholipase domain-containing 3
eIF2α	eukaryotic initiation factor 2α
Ptgs2	prostaglandin-endoperoxide synthase 2
*Qrich1*	glutamine-rich 1
ROS	reactive oxygen species
SEC11A	sec11 homolog A
Socs3	suppressor of cytokine signaling 3
SOD	superoxide dismutase
Spata21	spermatogenesis-associated 21
TEM	transmission electron microscopy
*Tjp1*	tight junction protein 1
Umod	uromodulin
UPR	unfolded protein response
Xbp1	X-box binding protein 1

## References

[1] Matthews H.D., Wynes S. (2022). Current global efforts are insufficient to limit warming to 1.5 °C. Science.

[2] Alexeeva N., Erbajeva M., Khenzykhenova F. (2015). Lasiopodomys brandti in Pleistocene of Transbaiklia and adjacent territories: Distribution area, evolutionary development in context of global and regional events. Quat. Int..

[3] De Toni L., Finocchi F., Jawich K., Ferlin A. (2023). Global warming and testis function: A challenging crosstalk in an equally challenging environmental scenario. Front. Cell Dev. Biol..

[4] Jiang G., Liu J., Xu L., Yu G., He H., Zhang Z. (2013). Climate warming increases biodiversity of small rodents by favoring rare or less abundant species in a grassland ecosystem. Integr. Zool..

[5] Ma L., Li H., Liu T., Liang L. (2019). Abrupt temperature change and a warming hiatus from 1951 to 2014 in Inner Mongolia, China. J. Arid Land.

[6] Hu Q., Pan F., Pan X., Zhang D., Li Q., Pan Z., Wei Y. (2015). Spatial analysis of climate change in Inner Mongolia during 1961–2012, China. Appl. Geogr..

[7] Shahat A.M., Rizzoto G., Kastelic J. (2020). Amelioration of heat stress-induced damage to testes and sperm quality. Theriogenology.

[8] Myers P., Lundrigan B.L., Hoffman S.M., Haraminac A.P., Seto S.H. (2009). Climate-induced changes in the small mammal communities of the Northern Great Lakes Region. Glob. Change Biol..

[9] Jiang G., Zhao T., Liu J., Xu L., Yu G., He H., Krebs C., Zhang Z. (2011). Effects of ENSO-linked climate and vegetation on population dynamics of sympatric rodent species in semiarid grasslands of Inner Mongolia, China. Can. J. Zool..

[10] Karna K.K., Shin Y.S., Choi B.R., Kim H.K., Park J.K. (2019). The role of endoplasmic reticulum stress response in male reproductive physiology and pathology: A review. World J. Men’s Health.

[11] Ji Y.-L., Wang Z., Wang H., Zhang C., Zhang Y., Zhao M., Chen Y.-H., Meng X.-H., Xu D.-X. (2012). Ascorbic acid protects against cadmium-induced endoplasmic reticulum stress and germ cell apoptosis in testes. Reprod. Toxicol..

[12] Karna K.K., Choi B.R., Kim M.-J., Kim H.K., Park J.K. (2019). The effect of Schisandra chinensis Baillon on cross-talk between oxidative stress, endoplasmic reticulum stress, and mitochondrial signaling pathway in testes of varicocele-induced SD rat. Int. J. Mol. Sci..

[13] Hosseini M., Shaygannia E., Rahmani M., Eskandari A., Golsefid A.A., Tavalaee M., Gharagozloo P., Drevet J.R., Nasr-Esfahani M.H. (2020). Endoplasmic reticulum stress (ER stress) and unfolded protein response (UPR) occur in a rat varicocele testis model. Oxidative Med. Cell. Longev..

[14] Ma B., Zhang J., Zhu Z., Bao X., Zhang M., Ren C., Zhang Q. (2019). Aucubin, a natural iridoid glucoside, attenuates oxidative stress-induced testis injury by inhibiting JNK and CHOP activation via Nrf2 up-regulation. Phytomedicine.

[15] Bai L., Zhang Y., Zheng C., Xu S., He Y., Yu G., Huang D., Huang Y., Li M., Xu C. (2023). Tanshinone IIA protects mouse testes from heat stress injury by inhibiting apoptosis and TGFβ1/Smad2/Smad3 signaling pathway. Cell Stress Chaperones.

[16] Bai D., Wan X., Li G., Wan X., Guo Y., Shi D., Zhang Z. (2022). Factors influencing range contraction of a rodent herbivore in a steppe grassland over the past decades. Ecol. Evol..

[17] Aldahhan R.A., Stanton P.G. (2021). Heat stress response of somatic cells in the testis. Mol. Cell. Endocrinol..

[18] Abd El-Emam M.M., Ray M.N., Ozono M., Kogure K. (2023). Heat stress disrupts spermatogenesis via modulation of sperm-specific calcium channels in rats. J. Therm. Biol..

[19] Teixeira M.B., Ferreira J.C.P., Codognoto V.M., Rossi E.S., Pupulim A.G.R., de Carvalho J.C., Rattes P.Z., Oba E., Navolar F.M.N., Di Santis G.W. (2025). Heat stress induced by testicular insulation for 24 or 48 h rapidly impairs epididymal sperm quality and reduces spermatogenesis in rams. Small Rumin. Res..

[20] Rizzoto G., Boe-Hansen G., Klein C., Thundathil J., Kastelic J. (2020). Acute mild heat stress alters gene expression in testes and reduces sperm quality in mice. Theriogenology.

[21] Tang Z., Yang Y., Wu Z., Ji Y. (2023). Heat stress-induced intestinal barrier impairment: Current insights into the aspects of oxidative stress and endoplasmic reticulum stress. J. Agric. Food Chem..

[22] Xiang J., Wan C., Guo R., Guo D. (2016). Is hydrogen peroxide a suitable apoptosis inducer for all cell types?. BioMed Res. Int..

[23] Zhao Z., Cai Y., Lin X., Liu N., Qin Y., Wu Y. (2024). The Role of Heat-Induced Stress Granules in the Blood–Testis Barrier of Mice. Int. J. Mol. Sci..

[24] Li X., Zhu J., Lin Q., Yu M., Lu J., Feng J., Hu C. (2022). Effects of curcumin on mitochondrial function, endoplasmic reticulum stress, and mitochondria-associated endoplasmic reticulum membranes in the jejunum of oxidative stress piglets. J. Agric. Food Chem..

[25] Fani G., La Torre C.E., Cascella R., Cecchi C., Vendruscolo M., Chiti F. (2022). Misfolded protein oligomers induce an increase of intracellular Ca2+ causing an escalation of reactive oxidative species. Cell. Mol. Life Sci..

[26] Casas-Martinez J.C., Xia Q., Li P., Borja-Gonzalez M., Miranda-Vizuete A., McDermott E., Dockery P., Quinlan L.R., Goljanek-Whysall K., Samali A. (2026). Adaptive ER stress promotes mitochondrial remodelling and longevity through PERK-dependent MERCS assembly. Cell Death Differ..

[27] You K., Wang L., Chou C.-H., Liu K., Nakata T., Jaiswal A., Yao J., Lefkovith A., Omar A., Perrigoue J.G. (2021). QRICH1 dictates the outcome of ER stress through transcriptional control of proteostasis. Science.

[28] Huang L., Cheng Y., Pan X., Li H., Liu J., Zhao G., Huang L., Wang L., Song C., Song P. (2026). DDIT3-Mediated Endoplasmic Reticulum Stress Drives Manganese-Induced Apoptosis in Bovine Testicular Leydig Cells. Cell Stress Chaperones.

[29] Cairrao F., Santos C.C., Le Thomas A., Marsters S., Ashkenazi A., Domingos P.M. (2022). Pumilio protects Xbp1 mRNA from regulated Ire1-dependent decay. Nat. Commun..

[30] Bartoszewska S., Sławski J., Collawn J.F., Bartoszewski R. (2023). Dual RNase activity of IRE1 as a target for anticancer therapies. J. Cell Commun. Signal..

