# Supplementary material for: Heat Stress-Induced Male Reproductive Dysfunction in Brandt’s Vole (Lasiopodomys brandtii): A Central Role for Endoplasmic Reticulum Stress
Source: Biology (Basel). 2026 Sep 5;15(17):1553. doi: 10.3390/biology15171553 (PMC13565436; doi:10.3390/biology15171553)
Supplement: Supplementary file 1 [file biology-15-01553-s001.zip › Figure S10 Original western blot images.pdf]

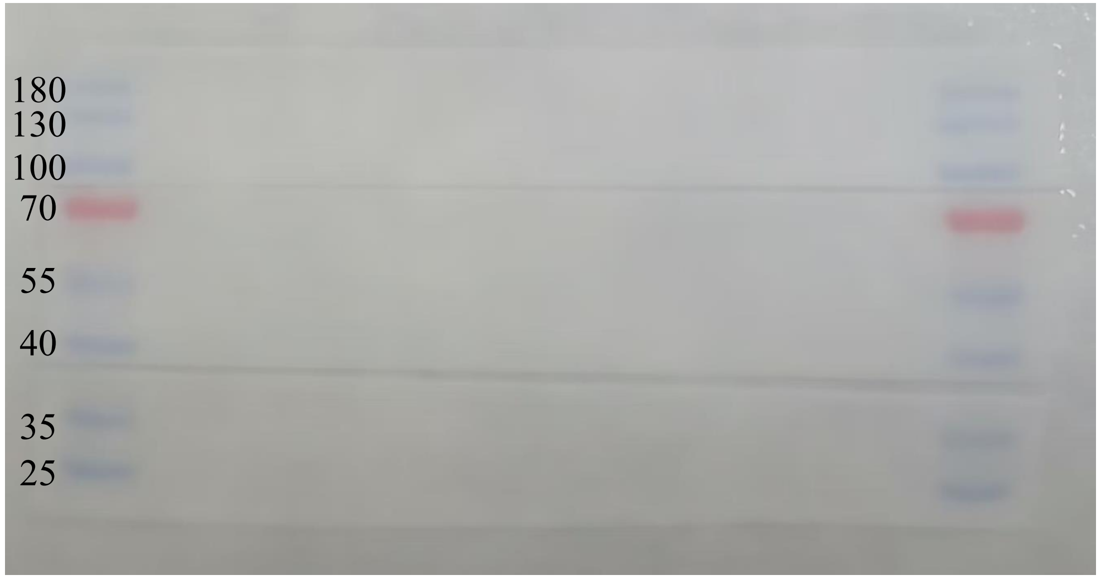
180
130
100
70
55
40
35
25

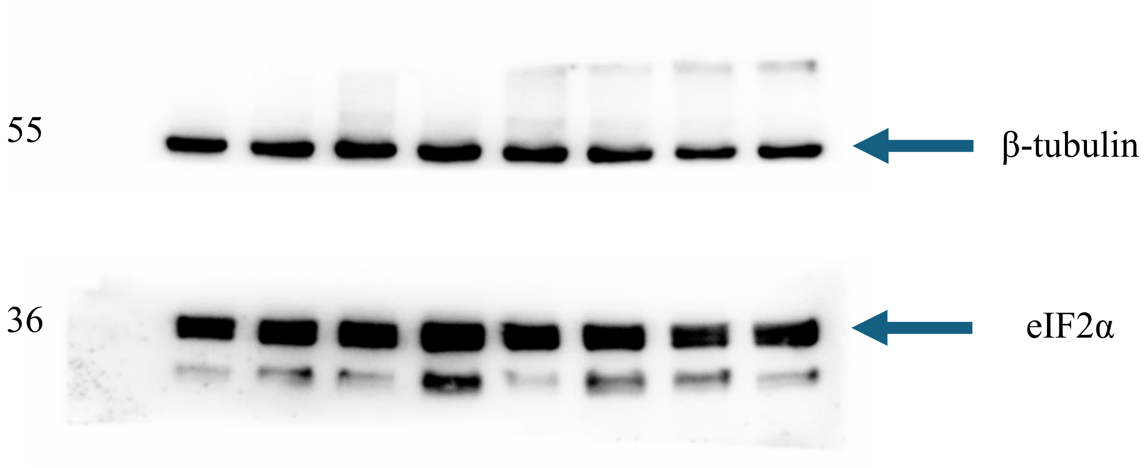
55
β-tubulin
36
eIF2α

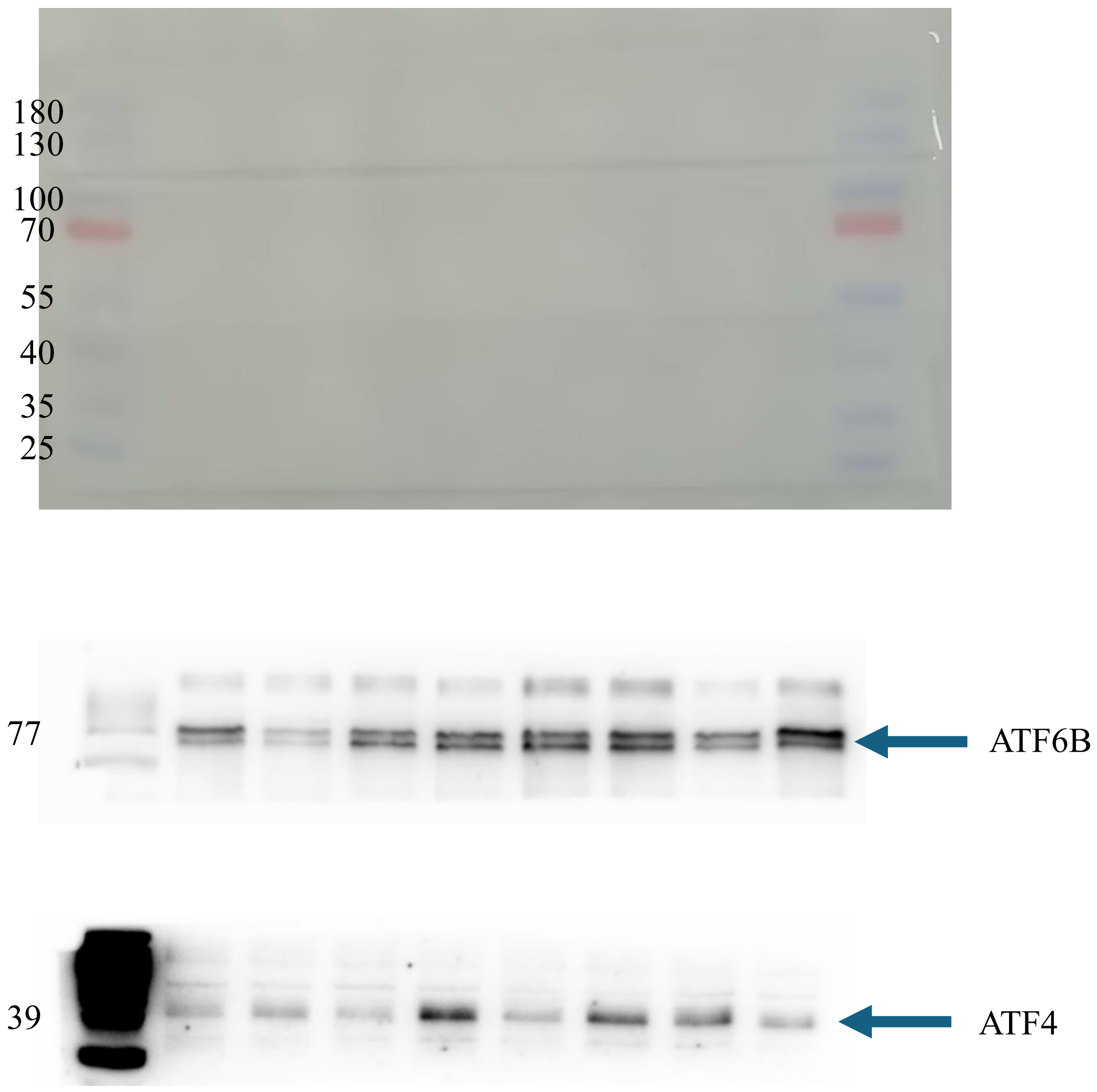
180
130
100
70
55
40
35
25
77
ATF6B
39
ATF4

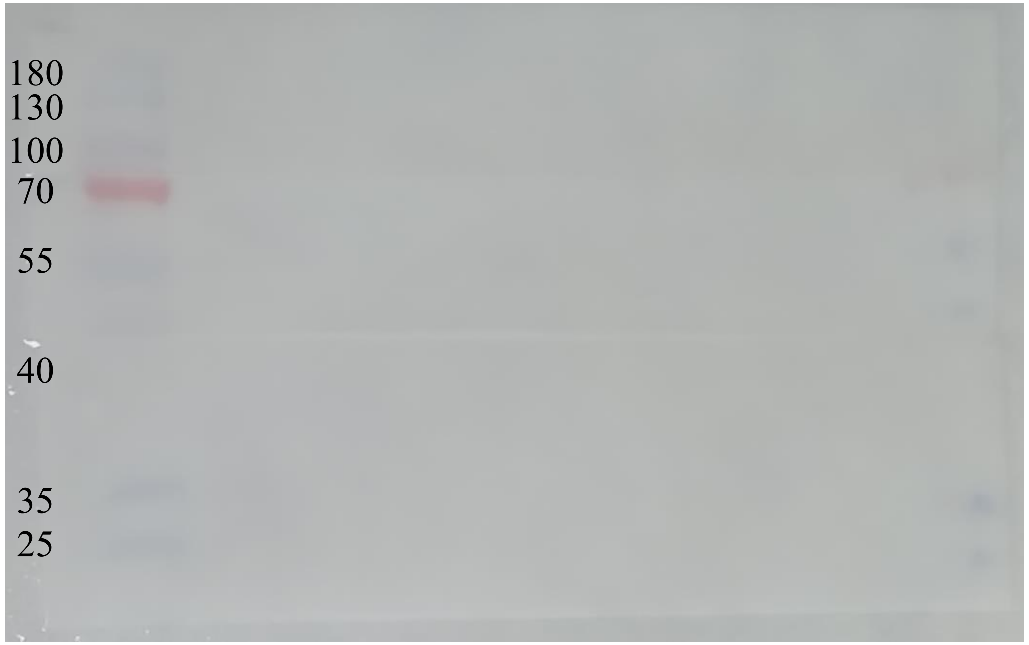
180
130
100
70
55
40
35
25

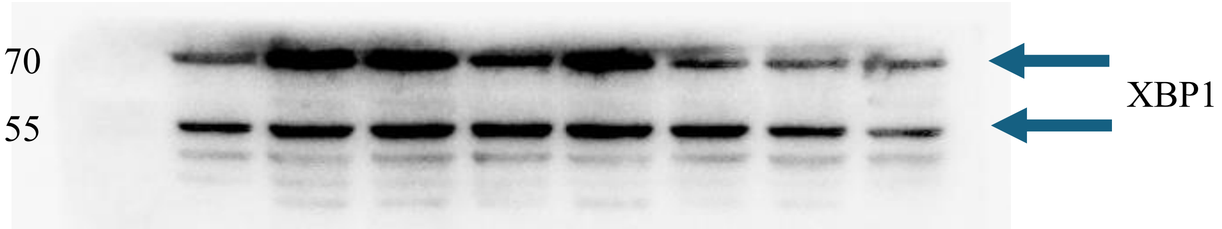
70
55
XBP1
