# Supplementary material for: Heat Stress-Induced Male Reproductive Dysfunction in Brandt’s Vole (Lasiopodomys brandtii): A Central Role for Endoplasmic Reticulum Stress
Source: Biology (Basel). 2026 Sep 5;15(17):1553. doi: 10.3390/biology15171553 (PMC13565436; doi:10.3390/biology15171553)
Supplement: Supplementary file 1 [file biology-15-01553-s001.zip › Supplementary figure legends.pdf]

Figure S1. Analysis of DEGs. Bar plot showing the number of up-regulated and down-regulated DEGs in C37, C39 and C41 groups compared to the Ctrl group (A). Venn diagram of DEGs across different comparisons (B). Volcano plot of DEGs in the Ctrl-vs-C37 (C), Ctrl-vs-C39 (D), and Ctrl-vs-C41 (E) comparison.

Figure S2. Gene ontology (GO) enrichment analysis of DEGs in pairwise comparisons of Ctrl and different exposed temperature. (A) Ctrl-vs-C37, (B) Ctrl-vs-C39, (C) Ctrl-vs-C41.

Figure S3. Kyoto Encyclopedia of Genes and Genomes (KEGG) analysis of DEGs in pairwise comparisons of Ctrl and different exposed temperature. (A) Ctrl-vs-C37, (B) Ctrl-vs-C39, (C) Ctrl-vs-C41.

Figure S4. Comparison of gene expression data between RNA-Seq and qRT-PCR.

Figure S5. Principal component analysis (PCA) of the proteomic profiles in the Ctrl and C39 groups.

Figure S6. Volcano plot showing the distribution of differentially expressed proteins (DEPs) between the Ctrl and C39 groups. DEPs were identified using a threshold of fold change ≥ 1.5 (or ≤ 0.67 for downregulation) and an unadjusted P-value < 0.05.

Figure S7. Subcellular localization of differentially expressed proteins (DEPs) in the Ctrl vs. C39 groups.

Figure S8. Overview of Gene Ontology (GO) functional annotation and enrichment analysis for significant DEGs. Panels show a summary of GO terms (A) and specific enrichment results for Biological Process (BP) (B), Cellular Component (CC) (C), and Molecular Function (MF) (D).

Figure S9. Bar chart illustrating the distribution of DEPs across major functional categories classified by KEGG level 2 annotations (A). Dot plot showing specific enriched pathways (level 3) identified in the comparison between the Ctrl and C39 groups (B). Butterfly plot detailing the differential enrichment of level 3 KEGG pathways for both upregulated and downregulated proteins (C).
