# Supplementary material for: Heat Stress-Induced Male Reproductive Dysfunction in Brandt’s Vole (Lasiopodomys brandtii): A Central Role for Endoplasmic Reticulum Stress
Source: Biology (Basel). 2026 Sep 5;15(17):1553. doi: 10.3390/biology15171553 (PMC13565436; doi:10.3390/biology15171553)
Supplement: Supplementary file 1 [file biology-15-01553-s001.zip › Supplementary tables.pdf]

Table S1 Sequences of primers used in this study.

Primer	Forward sequence (5'→3')	Reverse sequence(5'→3')
β-actin	ACATCCGTAAAGACCTCTATGCC	TACTCCTGCTTGCTGATCCAC
Aqp2	ACAACTACCTGCTGTTCCCCTC	TCCCAATCAGTGTCGGGCTCT
Atf3	TCTCCTTCCCGTTCAGTGACA	CAGTGCCATTGACATCCACCA
Atf4	CCTCGACCTAAACCTTATGACC	TTCTCCCTTGCCTTACGGAC
Ccdc33	CGTGTCTTCCACCCATACCAC	CAAGTTGCCCTTCCGAACCAC
Cebpd	ACTCCAAACGACCGATACCTC	ACAAATGTACCTTAGCTGCGAT
Cldn1	TACTCCTACGCTGGGGACAAC	CCTCCAGACACTTCATGCACT
Ddit3	CCCTGCCTTTCACCTTGGACA	TCCTCTTCGTTTCCAGCGGAT
Gli1	ATCTCCCTTTCTTGAGGTTGGCAT	CACTGGAGTTCCGTCTACCTCG
Hspa5	GCCATCCCGTGGTATAAACCC	GCCTCCCACAGTTTCAATACCAA
Hspb1	CGGAAATACACACTGCCTCCC	TCCACGGTAAGTGTGCCCTC
Iqub	GACCGATAAACTTCTCAAGCCA	TCACTATCACCGCATTCAGCC
Marchf10	ATCTACCAAACTACCTACGGCTA	CTTTCTCTGGCTTCTTTGGAGT
Mns1	CCAGGAACCAAATCGTGCAA	TGGCTTCTTCCATGTCTAGCTC
Ocln	CACGTTCGACCAATGCTCT	AATATGGCGATGCACATGACA
Pnpla3	GTGCAGTAACCTCATGTCTGT	TTGTCATCCCCACCATTCACA
Ptgs2	AAATCCTTGCTGCTCCAAC	TATAGTGCAGTTTTCCCCGTA
Qrich1	AACTGCACAAGAACAGACACC	GCCAGTCTGTTTTGAGCATCC
SEC11A	TGTCCCCTATGCCACACTCCA	GCAAGCACTTTACTGAGCCACCA
Socs3	CCTTTCTTATCCGTGACAGCTC	CCCTCACACTGGATGCGTA
Spata21	TCAGACACACAGGATCACCCC	GCTGCTCTTCTGAGGTCACAC
Tjp1	ACCCGAAACTGATGCTGTGGAT	GCTGGCTGGCTGTACTGTGA
Umod	CTATGCCACGCCCAGTAGCAA	TGAACAGAAAACCGGCCCTGA
Xpb1	TGACCACGTATACACCAAGCC	GCCCTCATAGCCACAGTCAC

Note: Aqp2, Aquaporin 2; Atf3, Activating Transcription Factor 3; Atf4, Activating Transcription Factor 4; Ccdc33, Coiled-coil domain containing 33; Cebpd, CCAAT enhancer binding protein delta; Cldn1, Claudin-1; Ddit3, DNA Damage Inducible Transcript 3; Gli1, GLI Family Zinc Finger 1; Hspa5, Heat Shock Protein Family A; Hspb1, Heat shock protein beta-1; Iqub, IQ motif and ubiquitin domain containing gene; Marchf10, Membrane Associated Ring-CH-Type Finger 10; Mns1, Meiosis specific nuclear structural 1; Ocln, Occludin; Pnpla3, Patatin-like phospholipase domain-containing 3; Ptgs2, Prostaglandin-Endoperoxide Synthase 2; Qrich1, Glutamine Rich 1; SEC11A, SEC11 homolog A; Socs3, Suppressor of cytokine signaling 3; Spata21, Spermatogenesis associated 21; Tjp1, Tight junction protein 1; Umod, Uromodulin; Xpb1, X-box binding protein 1.

Table S2 Results of RNA sequencing data filtering.

Samples	Total Reads	Clean Reads	Percentage of clean reads	Clean bases	GC Content	Q20	Q30
Ctrl_1	83,864,402	83,798,030	99.92%	12,448,096,028	50.4%	97.95%	93.64%
Ctrl_2	81,612,118	81,548,252	99.92%	12,131,568,000	50.31%	97.93%	93.58%
Ctrl_3	71,864,864	71,808,620	99.92%	10,675,469,464	50.5%	97.96%	93.67%
Ctrl_4	87,089,102	87,019,364	99.92%	12,934,862,392	50.66%	98.02%	93.86%
Ctrl_5	81,491,400	81,427,550	99.92%	12,125,702,808	50.37%	97.92%	93.55%
C37_1	91,923,760	91,851,644	99.92%	13,660,189,482	50.64%	97.94%	93.63%
C37_2	79,107,366	79,046,616	99.92%	11,755,914,264	50.69%	97.79%	93.15%
C37_3	82,283,432	82,218,256	99.92%	12,224,660,052	50.91%	98.0%	93.83%
C37_4	81,558,366	81,492,006	99.92%	12,117,329,656	50.58%	98.07%	94.04%
C37_5	83,441,688	83,374,172	99.92%	12,407,147,524	50.29%	98.01%	93.81%
C39_1	86,648,860	86,579,574	99.92%	12,893,319,196	50.61%	97.96%	93.7%
C39_2	82,760,986	82,695,958	99.92%	12,302,954,856	50.63%	97.94%	93.62%
C39_3	57,069,120	57,023,600	99.92%	8,489,560,582	50.8%	98.05%	93.96%
C39_4	82,488,35	82,425,00	99.92%	12,266,864,62	50.78%	97.92	93.55

	4	0		6		%	%
C39_5	80,748,588	80,682,482	99.92%	11,987,649,736	50.94%	98.14%	94.24%
C41_1	77,175,092	77,113,786	99.92%	11,464,940,588	50.64%	97.94%	93.63%
C41_2	89,062,916	88,991,976	99.92%	13,244,076,240	50.45%	97.94%	93.65%
C41_3	83,522,794	83,455,300	99.92%	12,418,915,564	50.62%	98.06%	93.96%
C41_4	69,970,310	69,916,992	99.92%	10,415,310,778	49.98%	97.87%	93.4%
C41_5	78,078,946	78,021,206	99.93%	11,626,628,794	50.19%	97.75%	92.97%

Note：Ctrl, control; C37, 37°C; C39, 39°C; C41, 41°C.

Table S3 Transcript assembly length distribution

Transcript length interval	200-500 bp	500-1 kbp	1 k-2 kbp	>2 kbp	Total
Number of transcripts	373111	120881	70119	109363	673474
Number of unigenes	373054	115203	49149	37172	574578
Number of coden genes	1065	5305	13690	60508	80568

Table S4 Read alignment to a reference sequence

ID	Total Reads	Mapped Reads (%)	Secondary alignments (%)	Unique alignments (%)
Ctrl_1	83,798,030	72,836,300(86.92)	49,982,753(59.65)	22,853,547(27.27)
Ctrl_2	81,548,252	70,929,610(86.98)	48,461,768(59.43)	22,467,842(27.55)
Ctrl_3	71,808,620	62,516,599(87.06)	42,826,533(59.64)	19,690,066(27.42)
Ctrl_4	87,019,364	76,029,959(87.37)	52,198,910(59.99)	23,831,049(27.39)
Ctrl_5	81,427,550	70,588,414(86.69)	48,286,322(59.3)	22,302,092(27.39)
C37_1	91,851,644	79,655,707(86.72)	54,434,132(59.26)	25,221,575(27.46)
C37_2	79,046,616	68,553,415(86.73)	46,679,832(59.05)	21,873,583(27.67)
C37_3	82,218,256	71,364,521(86.8)	48,893,540(59.47)	22,470,981(27.33)
C37_4	81,492,006	70,486,000(86.49)	48,325,093(59.3)	22,160,907(27.19)
C37_5	83,374,172	72,602,044(87.08)	49,652,150(59.55)	22,949,894(27.53)
C39_1	86,579,574	74,886,800(86.49)	51,181,146(59.11)	23,705,654(27.38)
C39_2	82,695,958	71,014,304(85.87)	48,193,761(58.28)	22,820,543(27.6)
C39_3	57,023,600	49,104,894(86.11)	33,324,539(58.44)	15,780,355(27.67)
C39_4	82,425,000	71,560,066(86.82)	48,867,532(59.29)	22,692,534(27.53)
C39_5	80,682,482	69,613,278(86.28)	47,245,644(58.56)	22,367,634(27.72)
C41_1	77,113,786	66,926,020(86.79)	45,714,308(59.28)	21,211,712(27.51)
C41_2	88,991,976	77,130,514(86.67)	52,416,553(58.9)	24,713,961(27.77)
C41_3	83,455,300	72,295,835(86.63)	49,572,677(59.4)	22,723,158(27.23)
C41_4	69,916,992	60,072,120(85.92)	40,527,396(57.97)	19,544,724(27.95)
C41_5	78,021,206	67,695,116(86.77)	46,071,849(59.05)	21,623,267(27.71)

Note：Ctrl, control; C37, 37°C; C39, 39°C; C41, 41°C.

Table S5 Unigenes annotations.

Databases Annotation	Number of Unigenes
Nr	63463
Swiss-Prot	60107
KEGG	40680
Gene_Ontology	57631
eggNOG	43964
Pfam	59484
CAZy	3271
SignalP	9130
TMHMM	12637

Table S6 Overview of protein and peptide identification and quantification.

Sample	Identified Peptides	Quantified Peptides	Identified Protein	Quantified Protein
Ctrl_1	67725	51407	5808	5298
Ctrl_2	67725	55594	5808	5465
Ctrl_3	67725	54412	5808	5446
C39_1	67725	54421	5808	5394
C39_2	67725	55591	5808	5531
C39_3	67725	55773	5808	5518
